# Biomimetic AgNPs@antimicrobial peptide/silk fibroin coating for infection-trigger antibacterial capability and enhanced osseointegration

**DOI:** 10.1016/j.bioactmat.2022.05.015

**Published:** 2022-05-20

**Authors:** Wenhao Zhou, Tian Bai, Lan Wang, Yan Cheng, Dandan Xia, Sen Yu, Yufeng Zheng

**Affiliations:** a Shaanxi Key Laboratory of Biomedical Metallic Materials, Northwest Institute for Non-ferrous Metal Research, Xi'an, 710016, China; b Department of Dental Materials, Peking University School and Hospital of Stomatology, Beijing, 100081, China; c School of Materials Science and Engineering, Peking University, Beijing, 100871, China

**Keywords:** Antimicrobial peptides, AgNPs, Antibacterial, Osteointegration, Titanium

## Abstract

Endowing implant surfaces with combined antibacterial and osteogenic properties by drug-loaded coatings has made great strides, but how to achieve the combined excellence of infection-triggered bactericidal and *in vivo*-proven osteogenic activities without causing bacterial resistance still remains a formidable challenge. Herein, antimicrobial peptides (AMPs) with osteogenic fragments were designed and complexed on the surface of silver nanoparticle (AgNP) through hydrogen bonding, and the collagen structure-bionic silk fibroin (SF) was applied to carry AgNPs@ AMPs to achieve infection-triggered antibacterial and osteointegration. As verified by TEM, AMPs contributed to the dispersion and size-regulation of AgNPs, with a particle size of about 20 nm, and a clear protein corona structure was observed on the particle surface. The release curve of silver ion displayed that the SF-based coating owned sensitive pH-responsive properties. In the antibacterial test against *S.aureus* for up to 21 days, the antibacterial rate had always remained above 99%. Meanwhile, the underlying mechanism was revealed, originating from the destruction of the bacterial cell membranes and ROS generation. The SF-based coating was conducive to the adhesion, diffusion, and proliferation of bone marrow stem cells (BMSCs) on the surface, and promoted the expression of osteogenic genes and collagen secretion. The *in vivo* implantation results showed that compared with the untreated Ti implants, SF-based coating enhanced osseointegration at week 4 and 8. Overall, the AgNPs@AMPs-loaded SF-based coating presented the ability to synergistically inhibit bacteria and promote osseointegration, possessing tremendous potential application prospects in bone defects and related-infection treatments.

## Highlights

• AMPs and AgNPs were complexed through hydrogen bonds to form a protein crowns structure.
• Silk fibroin matrix was able to maintain the activity of AMPs over 21 d and endow with the infection-trigger release.
• The functional coating achieved synergistic antibacterial properties by damaging membrane structure and generating ROS.
• The coating displayed acceptable osteogenic properties *in vitro* and observably promoted osteointegration *in vivo.*

## Graphical abstract

**Figure:**
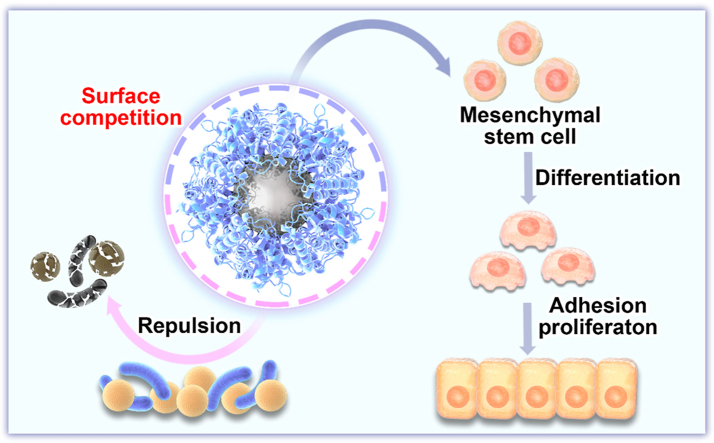

## 1 Introduction

Fixation devices or prosthetic joints used in orthopedic surgery are prone to biomedical device-related infection after implantation, with the incidence rate of 1–5%, and even 30% in the case of open fractures [1,2]. The problem of resistant bacteria induced by the abuse of antibiotics makes infection more complex [3], and this has a hugely adverse effect on osseointegration [4]. Therefore, how to endow the implant surface an efficient and long-term antibacterial function to inhibit bacteria has become the key to the success of the implantation operation [5]. Generally speaking, it is necessary to ensure that the implant surface is not invaded by bacteria, to ensure good contact between bone tissue and the material, and to inhibit infection while promoting osseointegration. Compared with the systemic use of antibiotics, changing the physical and chemical properties of the surface or directly constructing a drug-loaded coating can effectively ensure that the local concentration of the drug reaches the therapeutic level, while avoiding undesirable systemic effects [6]. Considering the frequent emergence of drug-resistant bacteria, there is an urgent need to actively explore alternatives to traditional antibiotics. For example, inorganic antibacterial agents (AgNPs) or antimicrobial peptides (AMPs) have a broad-spectrum bactericidal effect and are not easy to cause bacterial resistance, which has attracted widespread attention [7,8].

Compared with metal nanoparticles such as nano-gold and nano-zinc, silver nanoparticles (AgNPs) for surface modification of hard tissue implants to endow them with antibacterial ability show the following outstanding advantages [9]: AgNPs can lead to leakage of the contents by destroying the bacterial cell wall, inactive bacteria by binding to membrane proteins, RNA and genetic material, and can even react with a variety of enzymes including respiratory enzymes [10,11]. Benefiting from the complex antibacterial mechanisms introduced above, there are currently few reports on bacterial resistance to AgNPs [12]. Studies have shown that the minimum inhibitory concentration (MIC) of AgNPs against *Staphylococcus aureus* is 100 μg/mL [13]. Only when the working concentration exceeds this concentration value, AgNPs can interact with bacteria to kill bacteria, but this concentration usually leads to apoptosis of cells, so the concentration of AgNPs should be kept as low as possible [14]. To overcome this problem, AgNPs have been combined with other antibacterial molecules including antibiotics, enzymes, and other inorganic antibacterial agents, so as to show a synergetic sterilization phenomenon, described as “value-added” [15,16]. Although the combination of AgNPs with antibiotics [17], gold nanoparticles [18] or nano-copper [19] has made a huge breakthrough, the development of new composite antibacterial agents with higher biological safety and biological activity (promoting bone formation) still has great research significance.

Antimicrobial peptides (AMPs) provide an effective inspiration for solving the above problems. They are not only difficult to cause bacterial resistance, but also can gain multifunctionality through flexible amino acid sequence design [20]. It is generally believed that AMPs mainly induce bacterial death by destroying the bacterial cell membrane structure [21,22]. The main reason why AMPs can effectively avoid the adaptive mechanism of bacterial drug resistance is that AMPs destroy the cell membrane very quickly, and do not enter the bacteria inside, so the bactericidal effect can be achieved without activating adaptive immunity [23]. Although the amino acid sequences of AMPs can be flexibly designed, they generally have the characteristics of positive charge, small size and fast binding to cell membranes. More importantly, AMPs can aggregate on the surface of AgNPs, promote the dispersion of AgNPs through multivalent effect, and at the same time can exert a synergistic effect to show enhanced antibacterial effect. Lihong Liu et al. [24]constructed a cell-penetrating peptide to assist the reduction of silver nanoparticles and promote their uniform dispersion, and the novel nanocomposite antibacterial agent showed strong antibacterial effect and good biocompatibility. Mariana Vignoni et al. [25] reported that complexes of LL37 peptide with silver nanoparticles showed significantly reduced toxicity to primary skin cells, but significantly enhanced inhibition of wound infection. On the one hand, the synergistic effect with AMPs ensures that the dosage of AgNPs is greatly reduced, and the biosafety is significantly improved.On the other hand, it ensures long-term high-efficiency inhibition of infection and bacterial resistance. However, the antibacterial activity of AMPs is easily inactivated by environmental factors. Changes in typical *in vivo* environmental factors such as temperature and pH can cause AMPs degeneration, and the currently commonly used AMPs have insufficient osteogenic capacity.

As a natural active protein, SF is a very suitable orthopedic medical material with osteogenic ability [26]. Research shows that the structure of SF is highly similar with the structure of type Ⅱ collagen, which contributes to the generation of hydroxyapatite nucleation sites and promotes calcium deposition [27]. Besides, SF can inhibit the expression of Notch signals and promotes the expression of osteogenic genes [28]. Furthermore, tyrosine (Tyr) in the SF amino acid sequence has reducing ability, which can reduce silver ions *in situ* to obtain nanoparticles [29]. At the same time, it acts as a stabilizer to prevent the aggregation of nanoparticles and the inactivation of AMPs, with the advantages of maintaining antibacterial activity [30].

In this study, we designed AMPs with osteogenic fragments to form a protein crown structure on the surface of AgNPs, which was expected to achieve efficient synergistic antibacterial and osteogenic properties, and Ag@AP/SF coatings were constructed on Titanium (Ti). Complexes formed by AMPs and AgNPs played a synergistic effect, and SF was used to reduce silver ions *in situ* to obtain Ag nanoparticles and carry AgNPs@AMP. Firstly, the TEM was applied to observe protein crown structure and distinguish the difference between AgNPs and AgNPs@AMPs. Secondly, functional coatings co-cultured with *S.aureus in vitro* to verify the bactericidal and biofilm-inhibiting ability and to explore its sterilization mechanism. Finally, Ag@AP/SF coatings were co-cultured with BMSCs *in vitro* and implanted in rats’ femurs to verify their *in vitro* osteogenic ability and *in vivo* osseointegration ability, respectively.

## 2 Material and methods

### 2.1 Design and synthesis of chimeric peptides

The peptides (Table S1) were commercially synthesized (Sangon Biotech Co., Ltd., Shanghai, China) using the Fmoc (9-fluorenylmethyloxycarbonyl) method [31]. Designed antimicrobial peptide, NGIVKAGPAIAVLGEAAL-GGGGS named as amino peptides, and the new peptide sequence GGGGS-KRLFRRWQWRMKKY consisting of antimicrobial peptide JH8194 sequence (KRLFRRWQWRMKKY), named as carboxyl peptides. Both contained a flexible linker sequence (GGGGS), which could be used for efficient binding to the osteogenic segment in SF and promote the efficient expression of polypeptides in the bone microenvironment. HPLC and mass spectrometry were applied to ensure that the purity of the target peptide was above 98%, and PBS was used to prepare the peptide solution for the next reaction.

### 2.2 Preparation of silk fibroin-based coating

The key steps to purify silk fibroin include the dissolution of silkworm cocoon silk and the removal of sericin, which are carried out in accordance with established procedures [32]. In short, treatment with 0.5 wt% NaHCO_3_ aqueous solution at 100 °C for 30 min to remove sericin, then washed with distilled water, and dried at room temperature. After degumming, the mulberry silk fiber was dissolved in a 9.3 mol/L lithium bromide aqueous solution at 60 °C, and then dialyzed with a semipermeable membrane (MEMBRA-CEL, 12,000–14 000 MWCO) at room temperature for 72 h. The dialysate was centrifuged at 6000 r/min for about 5 min. The final concentration of the SF solution is about 5 wt%, and it is refrigerated at 4 °C for later use.

Adding AgNO_3_ (10–100 mM) and AMPs (0.2–0.8 g) to 2 mL of 5 wt% SF solution to obtain a clear SF/AgNO3/AMPs mixed solution with final AgNO_3_ and AMPs concentrations of 5–50 mM/L and 0.1–0.4 mg/L, respectively. The mixed solution was irradiated with UV lamp (40 W) for 0.5 h at room temperature, and AMPs assisted SF to reduce silver ions to obtain stable dispersed AgNPs. The cTi discs (10 mm × 10 mm × 0.5 mm) were polished with 800#, 1200# and 2000# sandpapers and then ultrasonically cleaned with acetone, ethanol and deionized water (DI). SF-based coatings were obtained by a simple spinning coating process. The silk fibroin coating alone carrying AgNPs, AMPs and simultaneous carrying AgNPs and AMPs were named Ag/SF, AP/SF and Ag@AP/SF, respectively.

### 2.3 Physical and chemical properties testes of SF-based coating

The topological structure and surface roughness were obtained by atomic force microscopy (AFM, DimensionICON, Bruker) in contact mode. The surface morphology and dispersion state of AgNPs were observed by transmission electron microscopy (TEM), scanning electron microscopy (SEM, S-4800, Hitachi, Japan) was used to observe the surface morphology of the coatings, and energy dispersive spectroscopy (EDS) was used for analyzing surface element distribution. X-ray photoelectron spectroscopy (XPS, Kratos, UK), microscopic infrared (FTIR) and X-ray diffraction (XRD) were applied to analyze the chemical composition and chemical binding of the surface. The hydrophilicity of the coating was determined using deionized water as the medium. Fluorescent bovine serum albumin (FITC-BSA) was used to test the protein adsorption capacity of the coating surface.

### 2.4 Ag ^+^ release into phosphate buffer solution (PBS)

To test the release profile of Ag^+^, the samples were placed in a 24-well plate, and 2 mL of PBS was added to fill each well, the pH of the PBS was adjusted to 7.4 and 5.0, and then placed in a 37 °C incubator. At the indicated time points, the PBS solution was collected and the Ag^+^ concentration was determined by inductively coupled plasma mass spectrometry (ICP-MS, Agilent 7700, USA), supplementing each well with fresh PBS buffer and continuing to incubation. In order to further verify the pH response characteristics of the SF-based coating, the pH of the immersion solution PBS was 7.4 for the first three days, and then the pH value was changed to 5.0 to continue the immersion, and the changes in the release rate of Ag^+^ were analyzed by ICP-MS.

### 2.5 Antibacterial properties

#### 2.5.1 Culture methods for gram-negative bacteria

Frozen stocks of *S.aureus* (ATCC 25922) were retrieved from the −80 °C freezer, and cultured in Luria Bertani Broth (LBB; Sigma Life Science, Sigma-Aldrich). Five milliliters of the frozen stock were cultured in 30 mL of the respective broth for 16 h in a shaker incubator (Benchmark Incu-shaker 10L, VWR) at 37 °C and 250 rpm. The concentration of *Staphylococcus aureus* determined by counting bacteria with a hemocytometer (Brightline, Hausser Scientific) and they were diluted to 1 × 10^6^ cells/mL in LBB.

#### 2.5.2 Antibacterial assay for planktonic and sessile bacteria

After the sample was co-cultured with the bacterial solution for a period of time, the number of bacteria adhering to the surface and in the suspension was tested by the plating method and WST reagent. Specifically, after the co-cultivation, the bacterial solution was collected and diluted with PBS, and then spread on the agar plate for colony counting. The test result was the release sterilization result. The samples were placed in 1 mL of PBS and ultrasonicated for 10 min, and then the PBS solution was coated on the agar plate for colony counting, and the test result was the contact sterilization result.

#### 2.5.3 SEM observation for bacteria

After co-culturing *Staphylococcus aureus* with the sample for a certain period of time, the bacterial morphology on the surface of the sample was observed by SEM. Specifically, the surface of the samples was washed twice with PBS, then fixed with 2.5% (v/v) glutaraldehyde (GA) and dehydrated in a series of ethanol (50–100%), and then the anchoring bacteria form were observed by SEM.

#### 2.5.4 Membrane permeability measurements

Propidium iodide (PI) (Invitrogen, USA) is used to measure the permeability of bacterial cell membranes because it can only be absorbed by bacteria with damaged membranes. The bacteria and the SF-based coating were co-cultured in LB medium pH 5.0 for 6 h, and then the bacteria on the coating surface were collected by ultrasound and mixed with PI in 0.85% NaCl (3 mL/L). After mixing thoroughly, incubate for 15 min at room temperature in the dark. Measure the fluorescence intensity in a 96-well plate with a microplate reader (SPECTRAMAX M5, MD, China), the excitation/emission wavelength is 488/630 nm, and the obtained intensity is normalized. After the bacteria and the sample were incubated in PBS at pH 5.0 for 6 h, the protein concentration in the bacterial suspension was determined by the Pierce BCA protein detection kit (Thermo Scientific, USA).

#### 2.5.5 Intracellular production of reactive oxygen species (ROS)

The generation of ROS in the bacteria was measured by DCFH-DA reagent. After the coating was co-cultured with the bacteria for 6 h, DCFH-DA reagent was added (final concentration 10 M) to react for 30 min. The bacteria were collected by centrifugation and resuspended in the same volume of PBS. The fluorescence intensity of the solution is detected by a microplate reader in fluorescence mode, which is excited and emitted at 488 nm and 525 nm, respectively.

### 2.6 BMSCs responses and *in vitro* biocompatibility of SF-based coatings

#### 2.6.1 Preparation of BMSC culture

BMSCs were extracted from the bone marrow cavity of the femur and tibia of three-week-old female Sprague-Dawley rats after euthanasia according to standard procedures [33]. In short, dissect the distal and proximal ends of the bone, use DMEM (Sigma-Aldrich) to flush the bone marrow out of the bone cavity and mix with 10% fetal bovine serum (FBS; HyClone, Logan, UT, USA) and 1% penicillin/streptomycin (P/S; Invitrogen, Grand Island, NY, USA). Use a filter to remove tissue debris and cell aggregates. The filtered BMSC is cultured in DMEM under standard cell culture conditions (37 °C, 5%/95% CO_2_/air, humidified, and sterile environment) to >75% converge. Subsequently, trypsin (Invitrogen) was used to isolate and passage BMSCs for biosafety testing of SF-based coatings.

#### 2.6.2 Direct culture of BMSCs with multi-functional coatings

Place the UV-sterilized samples in a standard 24-well cell culture plate, and then inoculate BMSCs directly on the surface of the sample at a density of 1 × 10^4^ cells/cm^2^, and culture in 1 mL DMEM under standard cell culture conditions. The positive control, called the "BMSCs" group, consists of BMSCs cultured in DMEM without adding samples to the wells. In order to simulate the condition of the circulation of the body to regularly remove the antibacterial agent released from the implantation site, the cell culture medium was collected regularly for ICP analysis and supplemented with 1 mL of fresh culture medium.

#### 2.6.3 SEM observation of BMSCs

After 24 h of culture *in vitro*, scanning electron microscope (SEM; Nova Nano-SEM 450, FEI Co., Hillsboro, OR, USA) was used to characterize the morphology of BMSCs on the coating surface. SEM sample preparation followed the standard procedure. The sample was taken out of the culture well and washed with PBS to remove non-adherent cells. The adherent cells were fixed with 2.5% glutaraldehyde for 1 h. After fixation, the surface of the sample was washed with PBS, and then dehydrated continuously in a gradient ethanol solution (50%, 75%, 90%, 2 × 100%; 10 min each time). Before SEM, platinum/palladium was sputtered (Model 108, Cressington Scientific Instruments Ltd., Watford, UK) at 20 mA for 40 s. An accelerating voltage of 20 kV was used to obtain SEM images and perform EDS analysis.

#### 2.6.4 BMSCs adhesion under direct cultures

The spreading state of BMSCs on the sample surface was observed by fluorescence staining. Specifically, the surface of the sample was gently washed twice with PBS to remove non-adherent BMSCs, and then fixed with 4% formaldehyde (10% neutral buffered formalin; VWR, Radnor, PA, USA) for half an hour, BMSCs were stained with nucleic acid stain (DAPI; Invitrogen) and Alexa Flour 488. Fluorescence pictures of BMSCs were taken using a fluorescence microscope (Nikon, Melville, NY, USA), and image J software was used for data acquisition of the fluorescence pictures to obtain data such as the average density and aspect ratio of cells.

#### 2.6.5 Ag ^+^ release behavior in direct culture with BMSCs *in vitro*

After the BMSCs were co-cultured with the samples for one day, the medium was collected and its pH value was measured using a calibrated pH meter to evaluate the effect of coating degradation on the stability of the medium, while using an inductively coupled plasma tester (ICP-OES; Optima 8000) test the concentration of Ag^+^ in the medium to evaluate the release behavior of Ag^+^. To minimize the influence of the medium in the ICP measurement, the collected medium solution was diluted at least 100-fold.

#### 2.6.6 Osteogenic differentiation of BMSCs

The ability of the SF-based coating to induce osteogenic differentiation of the BMSCs cells was evaluated by long-term co-culture of the samples with BMSCs cells. After 7 days and 14 days of co-cultivation, alkaline phosphatase detection kit was used to test the quantitative expression of ALP in BMSCs cells, and then BCIP/NBT alkaline phosphatase color development kit was applied to stain the surface of the samples for qualitative detection. After 28 days, the collagen on the surface was stained with 0.1% Sirius Red (Sigma, USA) saturated picric acid solution, and then treated with a destaining solution (0.2 M NaOH/methanol 1:1), and its absorbance value was measured at a wavelength of 450 nm as a quantitative indicator. After 28 days, the calcium nodules on the surface were stained with Alizarin Red S (ARS, Sigma; 2%, pH 4.3), and then decolorized with a calcium dissolving solution (10% cetylpyridinium chloride). Its absorbance value was measured at a wavelength of 450 nm as a quantitative indicator.

#### 2.6.7 Gene expression analysis

Real-time reverse transcriptase polymerase chain reaction (RT-PCR) was applied to assess the expression of osteogenesis-related genes in BMSCs. After 7 days and 14 days of co-culture, BMSCs cells were lysed by lysis buffer, and the released RNA particles were collected and converted into cDNA. Subsequently, the resulting cDNA was amplified and the primers (5′-3′) used in this paper were listed in Table S2. The housekeeping gene 18S rRNA was used to normalize the relative expression level of mRNA, and finally the gene expression level was determined by cycle threshold (Ct) value according to the ΔΔCt method. Set the parameters of the real-time PCR instrument as follows: preheating at 95 °C for 10 min, and 39 cycles of amplification (heating at 95 °C for 15 s, extension at 55 °C for 20 s, and annealing at 72 °C for 20s).

#### 2.6.8 Western blotting

Western blotting was used to assess protein expression levels in BMSCs. Specifically, after 10 days of co-cultivation, the BMSCs on the surface of the sample were collected with a cell scraper, ice bathed for half an hour, and then centrifuged at high speed (12,000 rpm) for 10 min, and the supernatant was collected as total protein. Specific primary antibodies (against ALP, COL-1, OCN, β-Actin, and Runx-2) were diluted 1:1000, and secondary antibodies were diluted 1:7000 in TBST buffer. The membrane was first reacted with the primary antibody (4 °C, overnight), and then with the secondary antibody (room temperature, 1 h), and finally the signal was detected with an enhanced chemiluminescence kit.

### 2.7 *In vivo* bone implantation

All animal experimental procedures were approved by the Ethics Committee of Peking University School of Medicine, Beijing, China (LA2019019). Twelve 8-week-old male Sprague-Dawley (SD) rats were used to assess the osseointegration of the samples (n = 6). The pure titanium rod was used as the control group, and the pure titanium rod modified by Ag@AP/SF coating was used as the experimental group. The rats were anesthetized by intramuscular injection of 1% sodium pentobarbital solution. Bilateral holes (Ф2 mm x 6 mm) were made on the femoral condyle, and the incision was sutured after inserting the sample.

New bone formation around the implant and fusion of the new bone with the implant interface were assessed by Micro-CT (Inveon MM CT; Siemens, Germany) at 80 kV. Inveon Research Workplace software (Siemens, Germany) was used to reconstruct 2D images into 3D images. Femurs were fixed in 10% neutral buffered formalin, washed with deionized water, dehydrated with graded ethanol, and embedded in methyl methacrylate. Then, slices with a thickness of 200 μm were cut along the vertical axis of the sample and then thinned to a thickness of 30–40 μm. Samples could be used for SEM observation and elemental analysis, and also for methylene blue and acid fuchsin staining. Organ samples of heart, liver, spleen, lung, and kidney were stained with hematoxylin and eosin (HE), and sections were observed by light microscopy (BX53, Olympus, Japan).

### 2.8 Statistical analyses

All experiments described above were repeated in triplicate and tested for homogeneity of variance. Parametric datasets were analyzed using one-way analysis of variance (ANOVA) followed by Tukey's HSD post hoc test. Statistical significance was considered at P-value < 0.05.

## 3 Results

### 3.1 Observation of AgNPs and AgNPs/AMPs complex

**Fig. 1:**
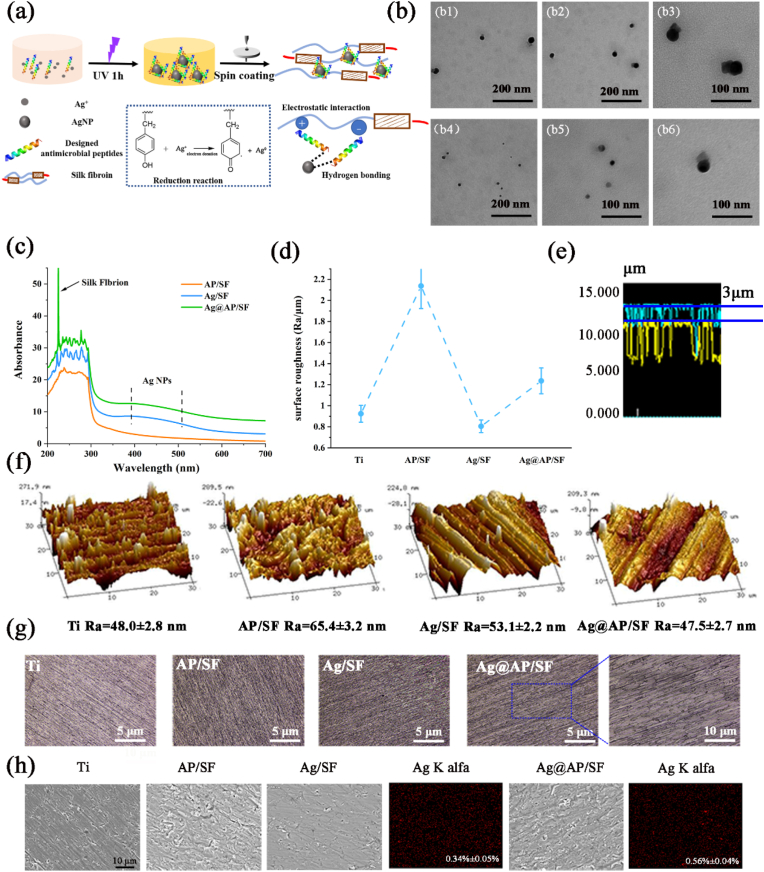
(a) The schematic diagram illustrated the formation of silver nanoparticles and the construction of a SF-based coating with antibacterial and bone-promoting functions; (b1-b3) AgNPs reduced by SF, (b4-b6) AgNPs reduced by SF/AMPs; (c)UV–vis spectra; (d) surface roughness; (e) layer thickness of Ag/Ap specimen; (f) AFM observation of the surface morphology; (g) surface morphology; (h) SEM observation and EDS analysis of the coated specimens.

As shown in Fig. 1a, with the aid of ultraviolet irradiation, because the tyrosine (Tyr) residues of SF were reductive, Ag ^+^ could be reduced *in situ* to obtain AgNPs, which were then spin-coated on the surface of the Ti basement, constructing a SF-based functional coating to endow it bactericidal and osteogenic functions. As shown in the TEM image of Fig. 1b, the AgNPs reduced by SF without the assistance of AMPs were spherical, with a particle size of about 50 nm. Aggregations formed between the particles, and the morphology changed with edges and corners, which were not conducive to bacteria-killing (Fig. 1b). However, the addition of AMPs effectively inhibited the agglomeration phenomenon and was beneficial to the dispersion of AgNPs. Unlike previously reported [15], the addition of antibiotics greatly improved the reduction efficiency of SF (about ten times). The addition of AMPs could only double the reduction efficiency of SF (n = 10), and the AMPs aggregated an obvious protein crown on the surface of AgNPs, contributing to the synergistic antimicrobial effects. This was consistent with reports in the literature [34], AMPs helped to the dispersion of AgNPs and they could be combined through a series of forces such as hydrogen bonds and metalloprotein interactions. As verified by the UV spectrum (Fig. 1c), the characteristic peak of SF was at 260 nm, and the characteristic peak of AgNPs was at 430 nm without an obvious red shift, indicating that the mixed liquid was stable.

### 3.2 The surface composition and morphology of functional coating

According to the previously established methods [35], the surface morphology and roughness of samples were observed with a 3D laser scanning microscope (VK-X150, Keyence). As shown in Fig. 1g, the surface morphology and roughness of different coatings were significantly different. The average surface roughness (Ra) of Ag/SF was about 0.8 μm, which was even slightly lower than that of bare Ti (0.96 μm). Apparently, the surface of the Ag/SF was flat and dense, and no micelles or nano-fibers caused by the SF conformational change were observed, implying that AgNPs did not affect the SF conformation. However, the roughness of the AP/SF surface increased sharply (2.16 μm), due to the electrostatic attraction and entanglement of the long molecular chains of SF and AMPs, which caused the viscosity of the AMPs/SF solution increasing significantly, and the poor fluidity of the solution during the preparation process, resulting in rising of the surface roughness. Obviously, there were evenly distributed black spots on the surface of the Ag@AP/SF, which meant that AMPs and AgNPs combined to form a complex. Consistent with speculation, the Ra of the Ag@AP/SF was reduced to 1.3 μm, which was conducive to protein adsorption and cell adhesion on. As shown in Fig. 1e, the thickness of the Ag@AP/SF was about 3 μm, with sufficient thickness as a drug-loading platform.

Although the roughness obtained by AFM (Fig. 1f) was different from the roughness under the optical test, the trend was basically the same. The surface morphology of the AP/SF was completely distinct from that of the Ag/SF, and its surface displayed a more obvious hump structure, and the surface roughness was relatively high. Some large granular protrusions could be observed on the Ag/SF surface, which was the result of nanoparticles agglomeration. In contrast, only the Ag@AP/SF owned a flat and compact surface, no obvious agglomerates, and low surface roughness, which were most suitable for the surface modification of titanium-based implants.

As shown in Fig. 1h, SEM characterized the surface morphology of the SF-based coating, which covered the entire surface of the Ti implant, thereby optimally exploiting the entire surface area of the substrate. On the surface of bare Ti, parallel scratches caused by polishing could be clearly observed. After being modified by the AP/SF, there was an entanglement of macromolecular chains, and the formed coating presented rough surface wrinkles and many defects, due to the high concentration of the antimicrobial peptide and silk fibroin mixed solution. For Ag@AP/SF, surface defects were significantly reduced, uniform and dense, the AgNPs were evenly distributed in the coating, and only a few cluster-like aggregations existed. Except for a few large AgNPs@AMPs complexes, the overall surface of the Ag@AP/SF was dense and uniform, with a silver content of only 0.56%, far higher than 3.4% of the Ag/SF, which ascribed to the AMPs improving the reduction efficiency of SF. According to the cross-sectional view, the Ag@AP/SF was tightly bonded to the Ti substrate. The thickness of the coating was about a few microns, which was consistent with the data scanned by the laser (Fig. S1).

### 3.3 Chemical properties, wettability and protein adsorption

**Fig. 2:**
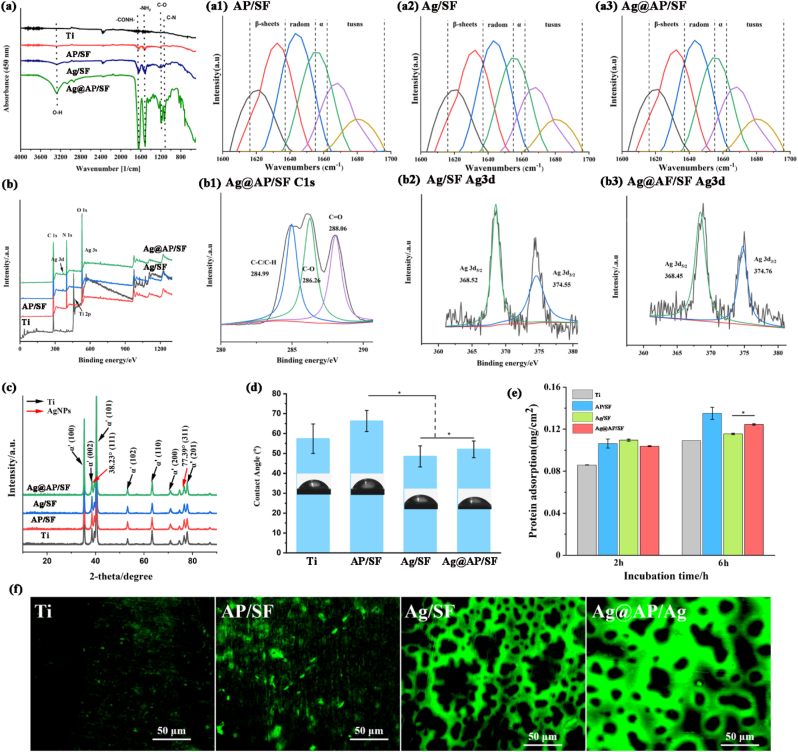
(a) FTIR spectra of bare and coated Ti specimens; (b) Survey spectra of XPS, and high resolution XPS spectra of (b1-b2) C 1s and (b3-b4) Ag 3d; (c) XRD spectra of bare and coated Ti specimens; (d) Contact angle; (e) Quantitative (2 h and 6 h) and (f) qualitative (6 h) testing of protein adsorption of SF-based coatings.

Since osteoblasts and bacteria will respond to surface chemistry, morphology, and hydrophilicity, and ultimately affect the success of the implant, it is important to fully understand the surface characteristics of the SF-based coatings. As shown in the ATR-FTIR spectrum (Fig. 2a), the characteristic peaks of the AP/SF and Ag/SF approximately overlapped, which reflected the good stability of SF as a coating matrix. In other words, AgNPs and AMPs did not cause changes in the molecular structure of SF. However, obvious characteristic peaks were observed on the Ag@AP/SF spectra at 1100, 1150, and 1300 cm^−1^, representing C–N stretching, C–O stretching and O–H bending, respectively. In addition, due to the formation of hydrogen bonds between the -COO^-^ and –NH_3_^+^ groups of AMPs and AgNPs, the secondary and tertiary protein structures changed, resulting in the appearance of primary, secondary or tertiary amine bands near 1200 cm^−1^. Combined with the appearance of a characteristic peak near 3250 cm^−1^ (hydrogen bond), it was confirmed that AMPs were firmly bonded to the surface of AgNPs through hydrogen bonds, forming complexes, which was consistent with the TEM results. In addition, the complexes not only stable the AgNPs, but also helped to expose the active end groups of AMPs. Moreover, in the characteristic spectra of AP/SF and Ag@AP/SF, characteristic peaks of amide groups were observed at 1630 cm^−1^ and 1550 cm^−1^, corresponding to amide I (–CONH–stretching) and amide II (-NH_2_ stretching), which meant that the combination with AgNPs did not cause degeneration of AMPs. Table 1 summarized the secondary structure content of different SF-based coatings. According to FSD spectra, Ag@AP/SF owned a higher secondary structure content of β-sheets and α-helices, contributing to stabilizing AgNPs/AMPs complexes and responding more sensitively to the microenvironment. The results of XPS (Fig. 2b) peak separations indicated that the Ag@AP/SF owned good chemical stability, and AgNPs had stable structures in both Ag/SF and Ag@AP/SF. In addition, XRD further confirmed the existence of hydrogen bonds between AgNPs and AMPs. As shown in Fig. 2c, due to the low content of antibacterial agents, the AgNPs in the Ag/SF and the AMPs in the AP/SF had no separate characteristic peaks. In sharp contrast, due to the formation of the complexes, the characteristic peaks of the Ag@AP/SF were significantly enhanced. In summary, it could be concluded that AgNPs and AMPs formed new stable complexes through strong hydrogen bonds, and load them into the SF matrix with an increase of β-sheets and α-helices contents, which was beneficial to the long-term maintenance of bioactivity.

**Table 1:** Secondary structure content of SF-based coatings.

	**β-sheets (** ± **1.6%)**	**random-coils (** ± **2.1%)**	**α-helices (** ± **1.8%)**	**Turns (** ± **2.4%)**
**AP/SF**	34.9	22.5	20.1	22.4
**Ag/SF**	21.8	26.0	12.6	39.6
**Ag@AP/SF**	33.2	25.4	16.9	22.4

Contact angle (CA) measurement was utilized to evaluate the wettability and a high CA value indicated that the surface was hydrophobic, while a low CA value presented that the surface was hydrophilic. The surface roughness and chemical groups have a great influence on the surface wettability, which will further affect the protein adsorption and cell adhesion. As shown in Fig. 2d, the surface of bare Ti was relatively hydrophobic (57.5°). In comparison, the Ag/SF decreased the surface CA value by 5° due to the hydrophilic α-helices structure in the SF molecular chain. Due to the high surface roughness, the CA value of the AP/SF surface reached 67.5°. The CA value of Ag@AP/SF was similar to that of bare Ti, and according to the verification of biological activity of titanium, implying that the Ag@AP/SF also had a certain biological activity. The protein adsorption experiment (Fig. 2e) displayed that the Ag@AP/SF owned significantly better protein adsorption capacity than other groups and its adsorption capacity would not decrease after 6 h. The quantitative results were different from the qualitative staining results (Fig. 2f), the protein adsorption values of Ag/SF and Ag@AP/SF were slightly lower than those of the AP/SF group, which was due to the different properties of the coating leading to a different binding ability to proteins. However, since Ag@AP/SF owned a stronger binding ability to protein, it was not easy to be eluted, so the detected quantitative value was low. After the material was implanted in the body, the first thing that occurred at the interface was the adsorption of protein. Studies have pointed out that the amount and type of protein adsorbed on the surface played a decisive role in the following adhesion and proliferation of cells. The protein covered the whole surface of Ag@AP/SF, implying excellent bioactivity.

### 3.4 Ag ions release curve

**Fig. 3:**
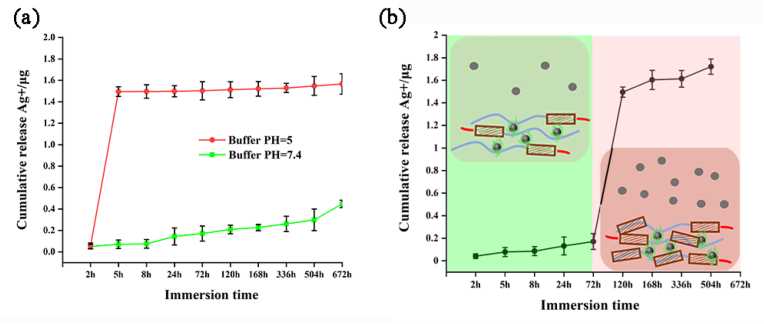
(a) The cumulative release proﬁle of Ag^+^ at different pH condition, and (b) pH-controlled release of Ag^+^ from Ag@AP/SF coating.

The initial burst release of antibacterial agents is one of the important reasons for the emergence of drug-resistant bacteria, which requires that an optimized drug-loaded coating should have the ability of intelligent release. As shown in Fig. 3a, the Ag@AP/SF owned obvious pH-responsive release characteristics. Under different pH conditions, the initial release amount was about ten times different (pH 5, 1.5 μg vs pH 7.4, 0.15 μg), and under the neutral conditions, it could maintain the long-term sustained release effect for 28 days. To further verify this conclusion, as shown in Fig. 3b, the Ag@AP/SF maintained stably in the neutral buffer for the first two days, and the release of Ag^+^ was at a low level when the buffer pH was reduced to 5.4 (simulating bacterial infection scenarios), a sharp increase in the release of Ag^+^ indicating the infection-trigger release behavior. As reported in the previous article [36], the change in the external pH environment would cause the transformation of the silk fibroin conformation, resulting in the transformation of the α-helices structure to the β-sheets structure, and the binding force on the AgNPs@AMPs complex was weakened, also the compactness of the coating was weakened, which eventually led to the rapid release of Ag^+^ to achieve infection-trigger antibacterial ability.

### 3.5 Antimicrobial abilities of SF-based functional coatings

**Fig. 4:**
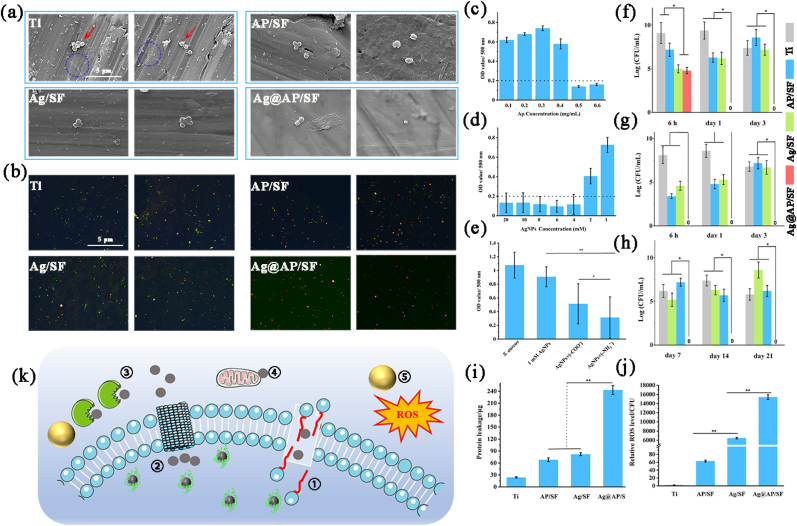
(a) SEM observation and (b) Live/Dead staining of bacteria morphology after cultured with samples for 24 h, blue circles marked the morphology of dead cells and the leakage of their contents; Quantitative assessment for antimicrobial activity of different groups against *S.aureus*: (c) the antibacterial ability of AP/SF specimen; (d) the bactericidal effects of Ag/SF specimen; (e) the synergetic effects of AgNPs/AMPs complex; Short-term (f) and long-term (g) killing effect against planktonic bacteria; (h) the short-term killing effect against adherent bacteria; (i) protein leakage and (j) Relative ROS level; (k) Schematic illustration of bacteria-triggered synergistic bactericidal mechanism of AgNPs/AMPs complex, 1 referred to AMPs/AgNPs complexes destroying the membrane structure, 2 referred to causing changes in ion channels, 3 referred to destroying the protein structure in the membrane, 4 referred to causing DNA denaturation, and 5 referred to generating reactive oxygen radicals.

The test of bacterial respiratory metabolites by WST-8 reagent reflected the antibacterial rate, which was applied to investigate the synergistic ability of AMPs and AgNPs. As shown in Fig. 4c, when the concentration of AMPs (amino antimicrobial peptides) exceeded 0.5 mg/mL, the absorbance of the bacterial solution was lower than the baseline of 0.2, which meant that most of the *S.aureus* were killed. Therefore, the minimum inhibitory concentration (MIC) of AMPs was 0.5 mg/mL. Meanwhile, when the AgNPs concentration exceeded 4 mM/mL, most of the bacteria in the solution were eliminated (Fig. 4d). In contrast, the antibacterial efficiency of AgNPs@AMPs complex against *S.aureus* was significantly higher than that of AgNPs or AMPs used alone. Finally, the AgNPs concentration of 1 mM/mL was chosen, at such a low dose, AgNPs owned almost no killing effect on *S.aureus*. However, when it was used with 0.2 mg/mL AMPs (much lower than MIC), the complexes exhibited a good synergistic antibacterial efficiency. We designed antimicrobial peptides with two different ends. Since the amino group or carboxyl group at the end of antimicrobial peptides would affect its charge in a liquid environment, which affected its binding to AgNPs and had a great impact on its synergistic antimicrobial ability, the experimental results also verified our conjecture. Obviously, for two AMPs with different end groups (-COO^-^ and –NH_3_^+^), the synergistic efficiency of amino AMPs with AgNPs was higher, and the clinical application value was greater (Fig. 4e). Therefore, amino AMPs were selected in this study. As shown in Fig. S2, the plate method directly reflected the synergistic antibacterial efficiency of AgNPs and AMPs. AgNPs of 1 mM/mL and AMPs of 0.2 mg/mL could completely kill the floating bacteria within 4 h. The bacterial solution was clear and transparent.

As shown in Fig. 4a, *S.aureus* maintained a completely spherical shape on the surface of pure Ti, and the bacteria rapidly aggregated to form small colonies, showing the tendency for rapid formation of biofilms. Both Ag/SF and AP/SF could damage the cell membrane of *S.aureus* and produce deformation, but its basic spherical shape could still be maintained. In sharp contrast, the permeability of the *S.aureus* cell membrane laid on the Ag@AP/SF was significantly changed, the cell membrane became wrinkled and defective, and the intracellular contents leaked out, in a dead state. Live/Dead staining was used to characterize the survival status of *S.aureus* (Fig. 4b). The green fluorescent dots represented live bacteria, and the red fluorescent dots represented dead bacteria. The *S.aureus* on the surface of pure Ti was basically live bacteria, with strong vitality. There were both live and dead bacteria on the surface of AP/SF and Ag/SF, and there was still a great risk of infection. In comparison, the *S.aureus* on the Ag@AP/SF was dead bacteria, not active, the number of bacterial corpses was small, and the surface cleanliness was maintained, with no tendency to form biofilms.

In this research, the WST-8 reagent method was used to quantitatively detect the antibacterial ability of the antibacterial coating, the short-term and long-term bactericidal effects of the Ag@AP/SF on *S.aureus* and the short-term antibacterial adhesion ability were studied in detail. As shown in Fig. 4f, compared with the bare Ti group, the number of bacteria on the AP/SF and Ag/SF was reduced by more than 100 times in a period of up to 24 h. However, the killing rate of AP/SF and Ag/SF dropped sharply on the third day, and almost completely disappeared in the following time (Fig. 4g). Moreover, their anti-adhesion ability disappeared completely on the third day (Fig. 4h), implying their limited antibacterial function and disappearing quickly with time. After 3 days, these surfaces were susceptible to bacterial infection and could not meet the clinical antibacterial requirements. In contrast, until the last time point (21 days), the inhibition rate of Ag@AP/SF on planktonic and adherent bacteria was maintained at least 10,000 times, which meant that Ag@AP/SF could not only inhibit bacteria adhesion, it could maintain high sterilization efficiency within one month after being implanted in the body, and there was no possibility of forming a biofilm on the surface.

As shown in Fig. 4i, the protein leakage level of the Ag@AP/SF was significantly higher, which meant that the cell membrane of the bacteria on its surface was severely damaged. Meanwhile, it was detected that the ROS content in the bacteria on the Ag@AP/SF was significantly higher than that of other groups, implying that the formation of AgNPs@AMPs complexes would promote the production of ROS (Fig. 4j), which was one of the main reasons for their efficient synergy. These results perfectly confirmed our hypothesis and a more detailed bacterial killing mechanism could be deduced (Fig. 4k).

### 3.6 Biocompatibility and osteo-differentiation of BMSCs

**Fig. 5:**
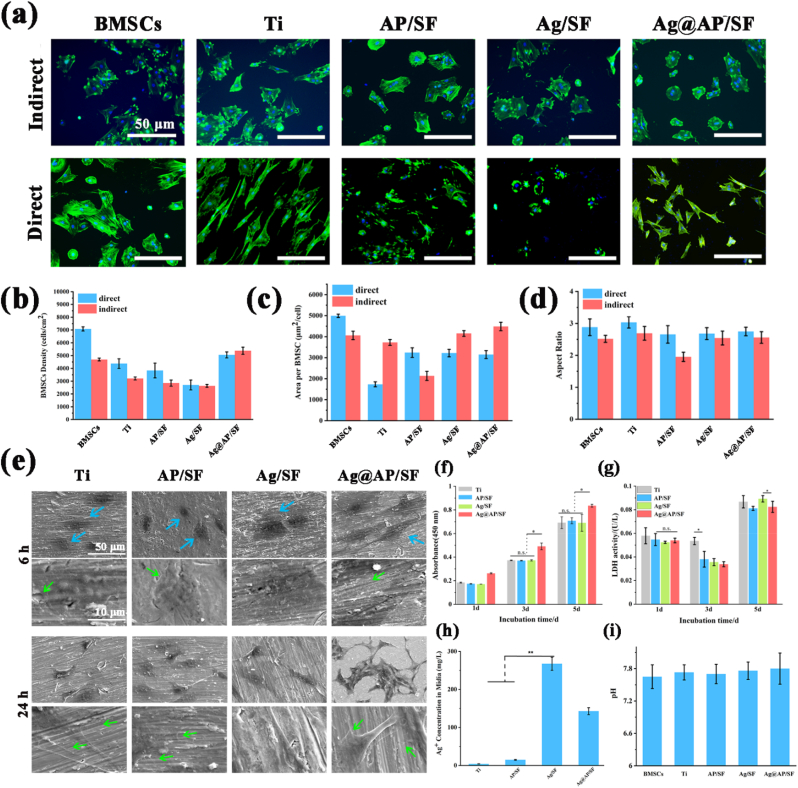
(a) Fluorescent staining of BMSCs directly cultured and indirectly cultured, blue indicated DAPI-stained nuclei, green indicated Alexa Fluor 488-stained F-actin; (b) Average density of BMSCs on the sample surface; (c) Average area of BMSCs on the sample surface; (d) The average aspect ratio of BMSCs on the surface of the sample; (e) The morphology of BMSCs was observed by SEM; (f) Cell viability test of CCK-8; (g) LDH test; (h) Silver ion concentration in culture medium; (i) pH of cell culture medium. Values are mean ± SD, n = 15P < 0.05.

Fig. 5a presented a representative fluorescence image of BMSCs directly attached to the surface (in direct contact) and there were two different types of cell morphology in the fluorescence image. Specifically, BMSCs with abundant pseudopodia and expanded morphology showed good adhesion, and BMSCs with round and non-expanded morphologies were considered to have poor adhesion. The number of BMSCs on the Ag@AP/SF had a larger spreading area and a good adherence state. In contrast, the adhesion state of BMSCs on the Ag/SF was slightly worse with the lightly elliptical shape, the unclear pseudopods, and the sporadic dispersion, indicating that AgNPs played certain toxicity to BMSCs and affected its adhesion state. However, the complexation with AMPs could effectively reduce the toxicity of AgNPs. From indirect contact fluorescence images of BMSCs, except for the Ag/SF, a large number of BMSCs adhered to the orifice plate around the sample and spread out. Interestingly, although the number of BMSCs around the Ag@AP/SF was small, BMSCs exhibited a unique interconnection morphology, indicating that the released Ag^+^ was not good for cell adhesion, but promoted cell-to-cell connections. In contrast, although the BMSCs around the AP/SF adhered well, the cells were scattered and there was no obvious cell connection. In a word, the Ag@AP/SF indirectly promoted the spreading of a large number of cells and the tight connection among cells presenting their optimized distribution state.

The adhesion density (dark blue) of BMSCs in direct contact with the sample was calculated quantitatively. As shown in Fig. 5b, the adhesion density of BMSCs on the Ag@AP/SF was higher than that of the Ag/SF and AP/SF, and slightly lower than that of the bare Ti group, which implied that the cytotoxicity level of the Ag@AP/SF was acceptable. The AP/SF was not conducive to the adhesion and proliferation of BMSCs on its surface, mainly due to its high surface adhesion, high toughness, and hydrophobic properties. Fig. 5b also displayed the adhesion density (light blue) of BMSCs in indirect contact with the sample. Obviously, the indirect contact cell density of the Ag/SF was the lowest. Interestingly, the indirect contact cell density of the Ag@AP/SF was slightly higher than that of the Ti group. Fig. 5c showed the average spread area (dark blue) of BMSCs in direct contact with the sample. In contrast, the spreading area of BMSCs on the surface of AP/SF and Ag@AP/SF was statistically significantly higher than that of Ag/SF coatings. BMSCs spread well on the Ag@AP/SF and showed potential proliferation and differentiation trends, while the cells that spread on the Ag/SF presented a smaller diffusion area and did not have long-term differentiation potential. Fig. 5d presented the average aspect ratio (dark blue) of BMSCs in direct contact with the sample. Obviously, the aspect ratio of the cells on the Ag/SF was the smallest (≈2), which was close to an elliptical shape, presenting an adverse effect on the proliferation of BMSCs. In contrast, the value of Ag@AP/SF was higher than that of the Ag/SF, which implied that AMPs would not affect the diffusion state of the cells, and effectively reduced the toxicity of AgNPs. As shown in Fig. 5e, it was obvious that the BMSCs were not spread well on the AP/SF and Ag/SF coating surfaces, with elliptical morphology and no filopodia. Conversely, the BMSC cells on the Ag@AP/SF presented a spindle shape, and the formation and abundance of filopodia could be observed within 6 h. With the contact time of up to 24 h, except for the Ag/SF cells were still dispersed and no filopodia, the BMSCs in the other group displayed a good spreading state, and abundant filopodia formed to perceive the coating's surface.

The result of CCK-8 was shown in Fig. 5f to reflect the cell viability. Since the amount of AgNPs was low, the Ag/SF did not show obvious cytotoxicity. The Ag@AP/SF was conducive to cell adhesion and proliferation, and the proliferation activity was significantly higher than that of the other three groups. This result also corresponded to the result of Live/Dead staining (Fig. S5). After 5 days of co-cultivation, there was no statistical difference in cell proliferation between the groups. LDH was applied to reflect the damage of the cell membrane of BMSCs (Fig. 5g). Obviously, the LDH value of the Ag/SF was slightly higher than that of the Ag@AP/SF, showing a damaging effect on the membrane structure of osteoblasts, but the complexation with AMPs would alleviate this damaging effect. Fig. 5h presented the Ag^+^ concentration in the medium after the BMSCs were co-cultured with SF-based coatings for 24 h. It was shown that Ag@AP/SF significantly slows down the release of Ag^+^. Only the rapid release of Ag^+^ causes the local high-concentration ion environment to be toxic to cells. In contrast, low concentrations of Ag^+^ are beneficial to cell proliferation and osteogenic differentiation [37]. Fig. 5i showed the pH value of the culture medium after the BMSCs were co-cultured with the sample for 24 h. There was no significant difference, and the overall range was between 7.6 and 7.8, which was suitable for the survival of BMSCs.

### 3.7 *In vitro* osteogenic differentiation test

**Fig. 6:**
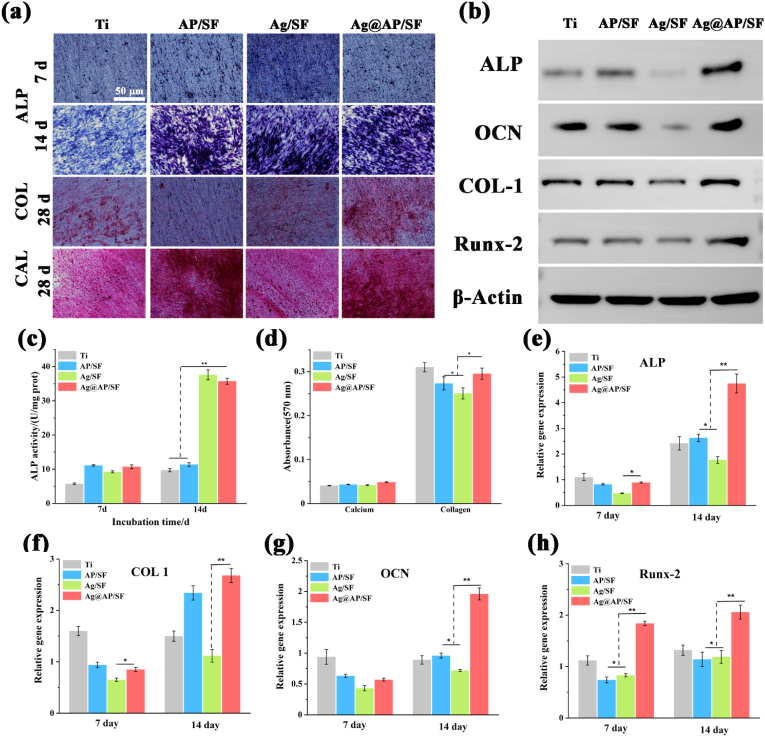
(a) Images of ALP staining, Sirius red staining and Alizarin red staining; (b) Western blotting; (c) Quantitative ALP expression of BMSCs on the surface of samples at 7 d and 14 d; (d) After 21 d of culture, quantitative analysis of collagen secretion and calcium deposition in the system; (e–h) Expression of osteogenesis-related genes in BMSCs on the surface of the samples, including ALP, COL 1, β-Actin, OCN, and Runx-2. *P < 0.05; ∗∗P < 0.01; **P < 0.001.

As shown in Fig. 6a, BMSCs on pure Ti, AP/SF, Ag/SF, and Ag@AP/SF were subjected to ALP staining for 7 d and 14 d and collagen (COL) for 28 d and calcium nodules (CAL) staining under the condition of osteoinductive medium, the corresponding quantitative statistics were shown in Fig. 6c and d. As an important indicator in the early stage of osteogenic differentiation, the expression of ALP reflected the trend of osteogenic differentiation of BMSCs. As shown in Fig. 6c, the ALP value of Ag@AP/SF was higher than other groups, and the difference was more obvious after 14 d of co-culture. At the same time, ALP qualitative staining was carried out. The darker the color, the stronger the expression of ALP activity. Consistent with the quantitative results, the expression of ALP on the surface of Ag@AP/SF was the highest. As typical markers of the late stage of osteogenic differentiation, collagen secretion (COL) and extracellular matrix mineralization (CAL) were treated with Alizarin Red (CAL) ARS and Sirius Red (COL) SR, respectively, after co-culture with BMSCs for 28 d. The red area on the surface represented the collagen and protein calcification secreted during the differentiation of BMSCs. Although darker stained areas were also observed on AP/SF and Ag/SF, the deepest red and largest stained areas appeared on the Ag@AP/SF surface, and the quantitative results were consistent with the staining images (Fig. 6d). The results of western blotting presented that the expression levels of ALP and COL-1 in Ag@AP/SF were significantly higher than those in other groups, and Ocn and Runx2 also showed significant up-regulation, indicating that AgNPs@AMPs-loaded coatings could activate the expression of canonical osteogenesis-related proteins. Bone-associated protein expression. Moreover, the expression of a series of osteoblast-related genes (*Alp*, *Col-Ⅰ* and *Ocn*) in the cells was also tested to reveal the osteogenic differentiation of osteoblasts co-cultured with different coatings (Figure e–h). After 7 and 14 days of co-culture, osteoblast-related genes were significantly up-regulated in Ag@AP/SF compared with other groups, and the expression level was the highest, indicating that the AgNPs@AMPs complexes were beneficial to the osteogenic differentiation of BMSCs.

### 3.8 *In vivo* studies for osseointegration

**Fig. 7:**
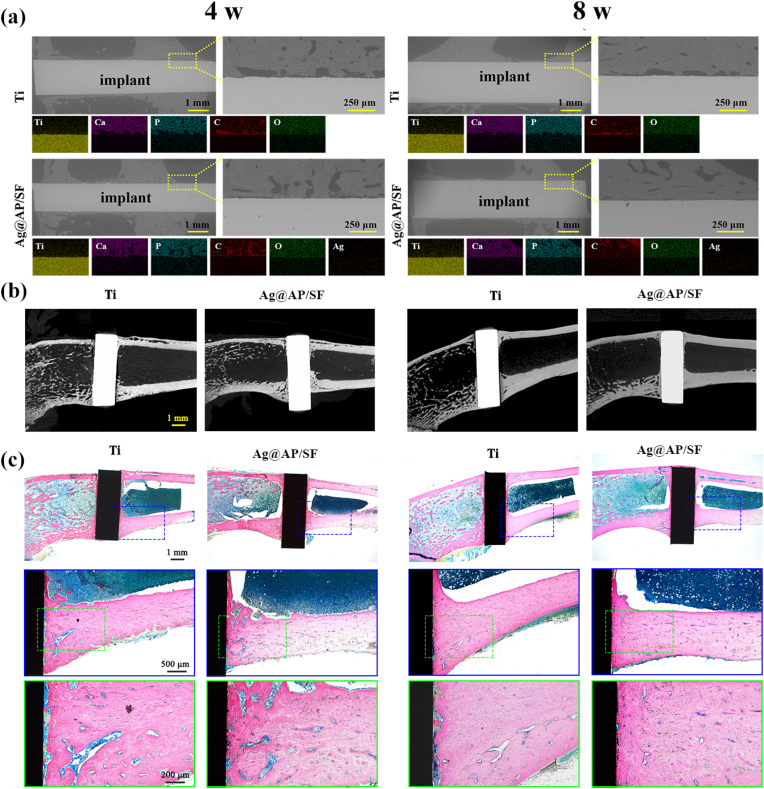
(a) SEM images and EDS images of the cross-sections of Ti rods and Ag@AP/SF coating-modified Ti rods implanted *in vivo* at 4 and 8 weeks; (b) Micro-CT imaging; (c) Light micrographs of hard tissue sections stained with methylene blue and acid fuchsin 4 and 8 weeks after implantation.

As shown in Fig. 7a, backscattered electron (BSE) images and EDS images were applied to analyze the cross-section of the orthopedic implants interface to study the variations in the composition and structure of the degraded layer. After implanting for 4 weeks, partial coverage of newly formed bone tissue appeared on the surface of pure Ti rods, mainly consisting of O, P, and Ca. However, the newly generated bone tissue on the surface of Ag@AP/SF increased significantly, indicating that the Ag@AP/SF could indeed induce new bone formation *in vivo*. At week 8, the new bone fully occupied the area around Ag@AP/SF (Fig. 7a). Additionally, the surface of the functional coating was closely contacted with the bone tissue. Conversely, discontinuous new bone tissue was rarely formed around the pure Ti rods. It has to be mentioned that the Ag@AP/SF did not degrade rapidly in the body and owned good stability, which could still be observed at week 8. Meanwhile, the element percentage of Ag in the EDS spectra of the Ag@AP/SF group at 4 W and 8 W was extremely low and almost undetectable, which meant that there was a very small amount of Ag released by the functional layer in the new bone, which played no adverse effects on bone healing. At 4 weeks and 8 weeks, the amount of new bone around the Ag@AP/SF was markedly increased compared to that of the bare Ti (Fig. 7b), and the new bone and implant interface tended to fuse, which contributed to the formation of a strong bone bond in the later stage. In summary, the Ag@AP/SF displayed a positive effect on the formation of new bone and promoted its fusion with the interface.

**Fig. 8:**
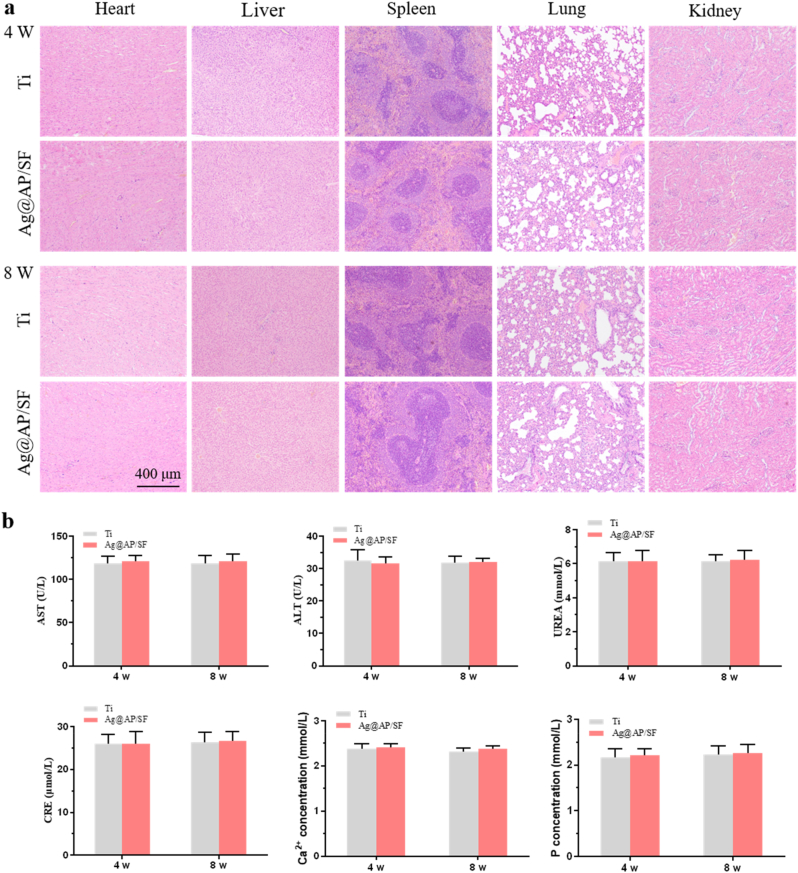
Biosafety tests of *in vivo* implantation. (a) Histological evaluations of organs; (b) Blood biochemistry test results. The data were expressed as mean ± standard deviation (SD).

New bone formation around the implant and healing at the new bone-implant interface was assessed by methylene blue and acid fuchsin staining of hard tissue sections (Fig. 7c). After 4 weeks of implantation, the bare Ti was surrounded by a thick layer of fibrous connective tissue and could not form good contact with the bone tissue. In sharp contrast, narrow, thin and discontinuous fibrous connective tissue appeared around Ag@AP/SF, and new bone appeared at the interface. After 8 weeks of bare Ti implantation, the fibrous tissue was significantly narrowed and partially replaced by new bone, which tended to combine with the implant surface. Surprisingly, the fibrous tissue around Ag@AP/SF disappeared completely and was replaced by newly generated bone tissue, and new bone was also detected in part of the pore structure, indicating that Ag@AP/SF not only promoted new bone formation but also promoted osseointegration at the interface. In addition, histological responses in rats were applied to assess the combined toxicity of Ag^+^ and AMPs release during metabolism and excretion (Fig. 8). The results displayed that no obvious pathological changes (tissue damage, inflammation, lesions, etc.) were found in the heart, liver, spleen, lung, and kidney after 4 weeks and 8 weeks, implying that the Ag@AP/SF coating could promote osseointegration without causing biosafety problems.

## 4 Discussion

### 4.1 Formation of Ag@AP/SF coatings

In this research, antimicrobial peptides (AMPs) with osteogenic fragments were designed and complexed on the surface of AgNPs. With the synergistic bactericidal effect of AgNPs and AMPs, the Ti implant was endowed with infection-trigger antibacterial and osteoinductive properties. AMPs aggregated on the surface of AgNPs to form a protein crown similar structure, which was confirmed by TEM observation (Fig. 1b). The hydrogen bonding force between AMPs and AgNPs was confirmed by FTIR (Fig. 2a). The protein crown structure owned the following advantages: (1) AMPs acted as a stabilizer to prevent AgNPs from agglomerating. The particle size of AgNPs reduced by SF was about 50 nm, and there was a tendency to agglomerate. With the aid of AMPs, the particle size of AgNPs was greatly reduced to 20 nm accompanied by uniform dispersion; (2) The combination with AgNPs was conducive to the exposure of the functional groups of AMPs and maximize its antibacterial ability; (3) The addition of AMPs improved the reduction efficiency of SF, which was mainly due to the electrostatic repulsion between SF and AMPs, leading to the more stretched conformation of SF, promoting the exposure of reducing terminal Tyr, so the reduction efficiency was significantly improved. The above analysis was consistent with recently published research that Liping Wang et al. [38] reported P-13 (a 13 amino acid peptide) protected AgNPs (P-13@AgNPs) with a hydrodynamic diameter of about 11 nm by a single-step reaction, where P-13 was favorable for the stability of the AgNPs solution. Mariana Vignoni et al. [39] capped silver nanoparticles with LL37 peptide to retain the bactericidal properties of silver nanoparticles with activities comparable to silver nitrate or silver sulfadiazine.

Easy to inactivate was the main reason limiting the application of AMPs. In this research, the use of SF as a drug-carrying matrix solved this thorny problem. As a natural biologically active protein, SF with abundant secondary structures of α-helices and β-sheets has been proven to maintain the activity of active molecules for a long time and act as ideal drug carriers [40]. Long-term antibacterial tests confirmed that the antibacterial activity of the SF-based coating could be maintained for up to 21 days (Fig. 4g and h), reflecting that the activity of AMPs could be maintained for more than three weeks. The characterization of the surface physical and chemical properties presented that the Ag@AP/SF was uniform and dense, with low roughness, good hydrophilicity, and protein adsorption ability, implying that the Ag@AP/SF was beneficial to the adhesion and proliferation of osteoblasts on the surface.

### 4.2 Antibacterial mechanism of Ag@AP/SF coating

The Ag@AP/SF displayed excellent anti-adhesion, planktonic-killing (up to 21 days), and biofilm formation inhibition effects (Fig. 4). According to Gristina's "surface competition" concept [41], if osteoblasts and proteins adhere to the surface of the implant before bacteria, they may cover the entire surface, effectively preventing bacterial invasion. However, once bacteria invade the surface of the implant to form a biofilm, it will cause serious postoperative infection and implant failure. This is why the surface of the implant demands long-lasting antibacterial ability. Benefitting from the complex bactericidal mechanism, AgNPs have high-efficiency broad-spectrum bactericidal ability and are not easy to cause bacterial resistance, but their potential biological toxicity limits their application prospects [42]. Recently, AgNPs are frequently combined with other antibacterial agents to play a synergistic bactericidal effect, which ensures efficient sterilization, and the biological safety is significantly improved. For instance, we previously reported that the synergistic use of AgNPs and antibiotics had increased the antibacterial efficiency by more than ten times [7], and the synergistic use of AgNPs and nano-copper showed an antibacterial period of more than one month [[43], [44], [45], [46]]. However, the unavoidable problem was that antibiotic-resistant bacteria and the biological safety of Ag would make the problem more difficult.

Benefiting from the advantages of a wide range of sources, low toxicity, high specificity, and designable structure, AMPs have become a very attractive new antibacterial agent and have great application prospects in the problem of implant infection [47]. Studies have shown that when AMPs are complexed with AgNPs, the resulting complex will have new properties, including enhanced antibacterial ability, no drug resistance, targeting effect, and less toxicity [[48], [49], [50], [51]]. However, the clinical application of AMPs still faces some difficult problems, such as biological toxicity caused by high doses, single antibacterial mechanism, insufficient efficacy against certain strains, poor stability, and easy inactivation in the presence of serum and proteolytic enzymes. In this study, the SF matrix was applied as a drug-load platform, achieving new infection-triggered bactericidal properties. This was due to the fact that SF was defined as a natural amphiphilic block copolymer consisting of hydrophobic (ordered, highly conserved) and hydrophilic (less arranged, rather complex) blocks mixed together to provide flexibility and stability to SF, so SF could stabilize the structure of AMPs and maintain their activity for a long time while providing microenvironmental responsiveness.

Since the concentration of AgNPs and AMPs was much lower than MIC, high efficiency and infection-trigger sterilization could only be attributed to AgNPs/AMPs complex and SF matrix, the specific antibacterial mechanism analyzed as follows (Fig. 4k): (1) AgNPs and AMPs were combined by hydrogen bonding force to form a protein crown-like structure, which was not only conducive to the formation of smaller size (20 nm) AgNPs, but also beneficial for aggregation and exposing functional end groups in AMPs to ensure maximum function; (2) SF matrix was pH-responsive due to its structural characteristics, and Ag^+^ release curve verified that acidic conditions would accelerate release, which ensured its intelligent response characteristics to the invasion of bacteria; (3) it caused serious damages to the structure of bacterial cell membrane. Because the phosphorothioate in the biofilm of *S.aureus* contained a large number of negatively charged phosphate groups, it could interact with the positive charged AMPs, and then caused bacterial membrane collapse and rupture [52], which was conducive to AgNPs entering the bacteria to perform functions. At the same time, AgNPs have also been reported to have the ability to destroy the membrane structure. SEM observations and protein leakage experiments (Fig. 4i) confirmed that the membrane structure of *S.aureus* was severely damaged; (4) AgNPs/AMPs complexes promoted the generation of ROS, which was one of the main bactericidal reasons, and its expression level was thousands of times higher than that of Ag/SF and AP/SF; (5) An important step in the antibacterial process of AgNPs was releasing Ag^+^, entering bacteria through interaction with thiol groups, destroying DNA structure, and affecting the synthesis of various proteins. Thanks to the permeability of bacterial membranes altered by AMPs, Ag^+^ was easier to enter and kill bacteria.

As shown in Fig. 4e, the study also revealed the synergistic efficiency of two kinds of AMPs with different end groups (–COO– and –NH_3_^+^) and AgNPs, confirming that amino AMPs played higher synergistic efficiency with AgNPs. The mechanism was inferred as follows: (1) Since both amino groups and Ag^+^ were positively charged, they were easily absorbed by the Tyr end groups in the SF molecular chain, so the efficiency and probability of the combination of amino AMPs and AgNPs were relatively high; (2) Amino groups of AMPs and AgNPs was easier to form hydrogen bonds, so the density of AMPs on the surface of the complex was higher, exposing more effective bactericidal fragments; (3) The bacterial membrane surface was negatively charged, and it was easier to interact with positively charged amino groups and Ag^+^, conducive to synergy. Because AgNPs had bactericidal activity against a variety of drug-resistant bacteria including methicillin-resistant *Staphylococcus aureus* (MRSA), AMPs could also be designed to target drug-resistant bacteria [53]. Therefore, this potential method to solve the problem of bacterial resistance showed broad prospects.

### 4.3 *In vitro* osteogenic performances of Ag@AP/SF

**Fig. 9:**
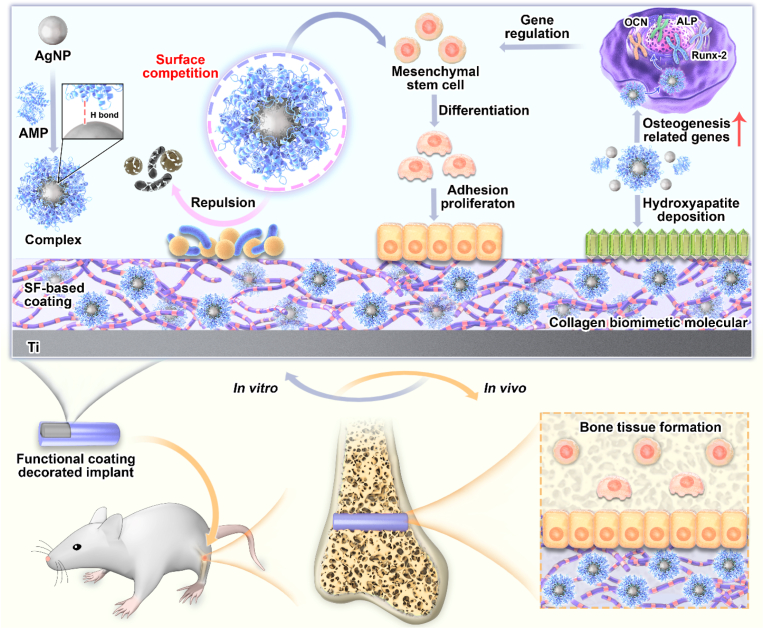
Scheme illustration of antibacterial mechanism and osteogenesis mechanism of SF-based functional coating.

While the Ag@AP/SF imparted the antibacterial ability to the implant, it also offered biocompatible and osteogenic properties to the Ti basement and supported the adhesion and spreading of BMSCs. Both the SF polymer matrix and the AMPs were protein-like long-chain molecules, with a three-dimensional structure and flexible segments. Designed AMPs with osteogenic segments not only owned the ability to induce osteogenic differentiation of BMSCs but also the protein crown structure formed on the surface of AgNPs could regulate its degradation behavior, suppressing the release rate and local concentration of Ag^+^. However, the ICP test of the culture medium (Fig. 5h) showed that about 140 μg/mL of Ag^+^ was released into the microenvironment within the first 24 h, but the current concentration was only local in one well of a 24-well plate (*in vitro* results), while the flow of body fluids and complex internal environment would lead to Ag^+^ in the implanting process was much lower than 140 μg/mL. As reported [18], the low-concentration Ag^+^ microenvironment contributes to the osteogenic differentiation of BMSCs. Although the specific underlying mechanism is still unclear, the expression of a variety of osteogenesis-related gene signals is up-regulated. Benefiting from choosing SF as the matrix, in addition to natural biological activity, and flexible and controllable processing properties, SF could be served as a stabilizer to steady AgNPs@AMPs complexes [54]. Moreover, maintaining the activity of AMPs for a long time greatly increased the bioactivity of SF-based coatings [55,56]. However, SF has an α-helical structure to facilitate the adhesion of osteoblasts, which greatly compensates for the toxicity of AgNPs. The average density difference of BMSCs between AP/SF and Ag@AP/SF on day 1 was less than 20%, and the density value of Ag@AP/SF was very close to the bare Ti group, which meant the cytotoxicity level of Ag@AP/SF within the acceptable range. Even in the initial burst release stage (within 24 h), the formation of AgNPs@AMPs complexes resulted in a significant decrease in the local concentration of Ag^+^. The pH response characteristic of Ag@AP/SF imparted by the SF matrix was also of great help to the controllable release of Ag^+^ and the biological safety. As show in Fig. 9, the experimental results displayed that the Ag@AP/SF greatly promoted the ALP expression, collagen secretion, mineralization deposition, and osteogenic gene expression of the BMSCs cells on the surface, indicating that the cells exhibited a good osteogenic differentiation trend, which played a very good expectation and verification for osteogenesis *in vivo*.

### 4.4 *In vivo* osteointegration of Ag@AP/SF

*In vivo* implantation experiments confirmed that the strength of the Ag@AP/SF on the surface of the titanium implant was sufficient to withstand changing stress and friction (within 8 W), which effectively guaranteed the Ag@AP/SF played its biological function in the formation of entire new bone and the bone healing process (Fig. 9). In addition, compared with bare Ti rods, the modification of Ag@AP/SF was the best choice for rapid osseointegration, partly thanks to the osteogenic properties of the SF matrix. The structure of SF is highly similar to the important components of bone matrix, collagen protein, leading to inherent remarkable bone regeneration properties of SF matrix [57,58]. Additional studies have shown that SF-based biomaterials, including functional coatings, hydrogels, and 3D printed scaffolds, have obvious characteristics of promoting new bone formation and bone fusion, and are one of the most ideal natural biomaterials for orthopedics [59,60]. Interestingly, when directly compared *in vivo*, SF and commercial Bio-Gide® collagen membranes had similar efficacy for guided bone regeneration (8.75 ± 0.80 and 8.47 ± 0.75 mm) in critical-sized rats 8 weeks after implantation of skull defect [61]. It was demonstrated that native *B. mori* SF scaffolds without any pre-seeded osteogenic cells, showed insufficient bone regenerative potential required for complete *in vivo* healing of large femoral defects in mice [62]. The design of the molecular structure of AMPs was one of its biggest features. By introducing molecular segments or structures with bone-promoting properties, the bone affinity properties of biomaterials can be greatly improved [[63], [64], [65]]. The AMPs segment in this study added molecular segments that were beneficial to osteogenesis, which was also one of the important reasons for the fusion of new bone and the coating surface *in vivo*. Moreover, the combination of low concentration AgNPs could also accelerate the new bone formation and osseointegration through the regulation of BMSCs behavior described above. Additionally, studies have pointed out that silver nanoparticles can stimulate fracture healing by attracting BMSCs, inducing BMSCs proliferation, and inducing osteogenic differentiation of BMSCs [66,67]. Therefore, the Ti-based implant modified with Ag@AP/SF was expected to perform a successful implant that could achieve rapid osseointegration.

Taken together, the *in vitro* and *in vivo* results have proved that designed AMPs/AgNPs-complexes were a promising approach to endowing the bioactivity and osseointegration ability of Ti-based implants. Further studies will be taken to advance the evaluation of the long-term bone ingrowth effects and *in vivo* mechanical strength of implants modified with bioactive Ag@AP/SF to provide more convincing and valuable experimental data for clinical translation.

## 5 Conclusion

In this study, based on the synergistic effects of AMPs and AgNPs, SF with an extremely high osteoinductive ability and high β-sheets content was used as coating matrix to simultaneously deliver AgNPs and AMPs, then applied by a spin-coating on bare Ti basement. The SF-based coating displayed better hydrophilic and protein-adsorption abilities. Due to the formation of AgNPs@AMPs complexes and the synergetic effects, the SF-based coating displayed outstanding anti-adhesion, planktonic-killing, and biofilm-inhibition abilities. Compared to untreated Ti, the Ag@AP/SF coating presented enhanced cell adhesion, proliferation, and osteogenic ability of BMSCs. Moreover, *in vivo* implantation studies verified that Ag@AP/SF coating promoted bone formation and healing. Therefore, our study demonstrates that the Ag@AP/SF with high-efficient bactericidal and osteogenic capacities hold great potential as a surface-modified coating for orthopedic implants.

## Declaration of competing interest

The authors declare that they have no known competing ﬁnancial interests or personal relationships that could have appeared to inﬂuence the work reported in this paper.

## CRediT authorship contribution statement

**Wenhao Zhou:** Conceptualization, Investigation, Data curation, Writing – original draft. **Tian Bai:** Investigation, Writing – original draft, Data curation. **Lan Wang:** Investigation, Data curation. **Yan Cheng:** Investigation. **Dandan Xia:** Investigation, Data curation. **Sen Yu:** Supervision, Resources, Writing – review & editing. **Yufeng Zheng:** Supervision, Resources, Writing – review & editing.

## Declaration of competing interest

The authors declare that they have no known competing financial interests or personal relationships that could have appeared to influence the work reported in this paper.
