# Supplementary material for: Biomimetic AgNPs@antimicrobial peptide/silk fibroin coating for infection-trigger antibacterial capability and enhanced osseointegration
Source: Bioact Mater. 2022 May 20;20:64–80. doi: 10.1016/j.bioactmat.2022.05.015 (PMC9127278; doi:10.1016/j.bioactmat.2022.05.015)
Supplement: Multimedia component 1 [file mmc1.docx]

Table S1. Details of antimicrobial peptides.

| **Number of**  **amino acids** | ***Peptide sequence**  **(N term to C term)** | | **Purity** | **Other modifications** |
| --- | --- | --- | --- | --- |
| 19 | | GGGGS-KRLFRRWQWRMKKY | 98% | none |
| 24 | | NGIVKAGPAIAVLGEAAL- GGGGS | 98% | none |

Table S2. Primer sequences used for RT-PCR analysis.

| **Gene** | | **Forward primer** | | **Reverse primer** |
| --- | --- | --- | --- | --- |
| **18S rRNA** | 5’-GTAACCCTTGAACCCCATT-3’ | | 5’-CCATCCAATCGGTAGTAGCG-3’ | |
| **ALP** | 5’-CATTCCCATTTCACATT-3’ | | 5’-AGTGAAGGGCTTCTTGTCTGTGT-3’ | |
| **COL 1** | 5’-TGATGCCAATGTGGTTCGTG-3’ | | 5’-TTGGTTGGGGTCAATCCAGTA-3’ | |
| **OCN** | 5’-CCAGCGTCAGAGTCCAGC-3’ | | 5’-GGACTGGGGCTCCCAGCCAT-3’ | |


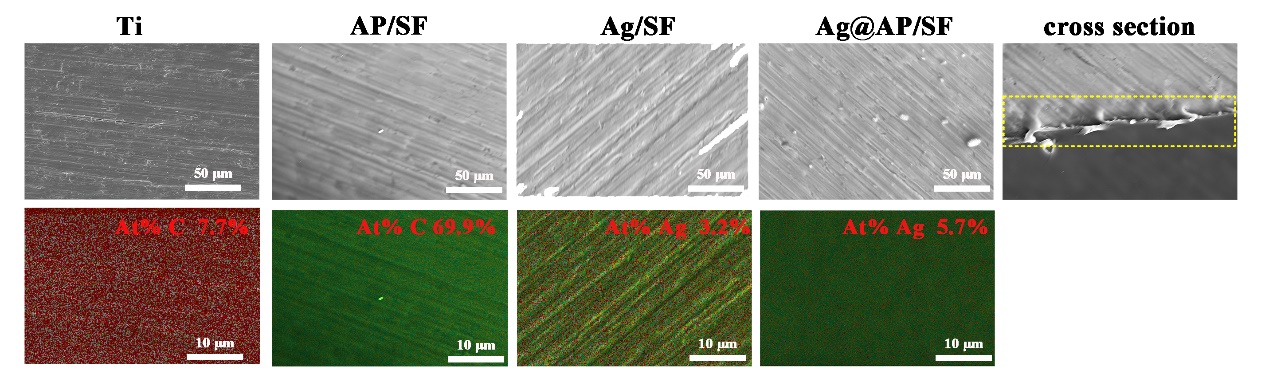


Figure S1. SEM and EDS observation of the surface morphology and cross-section of the coating.


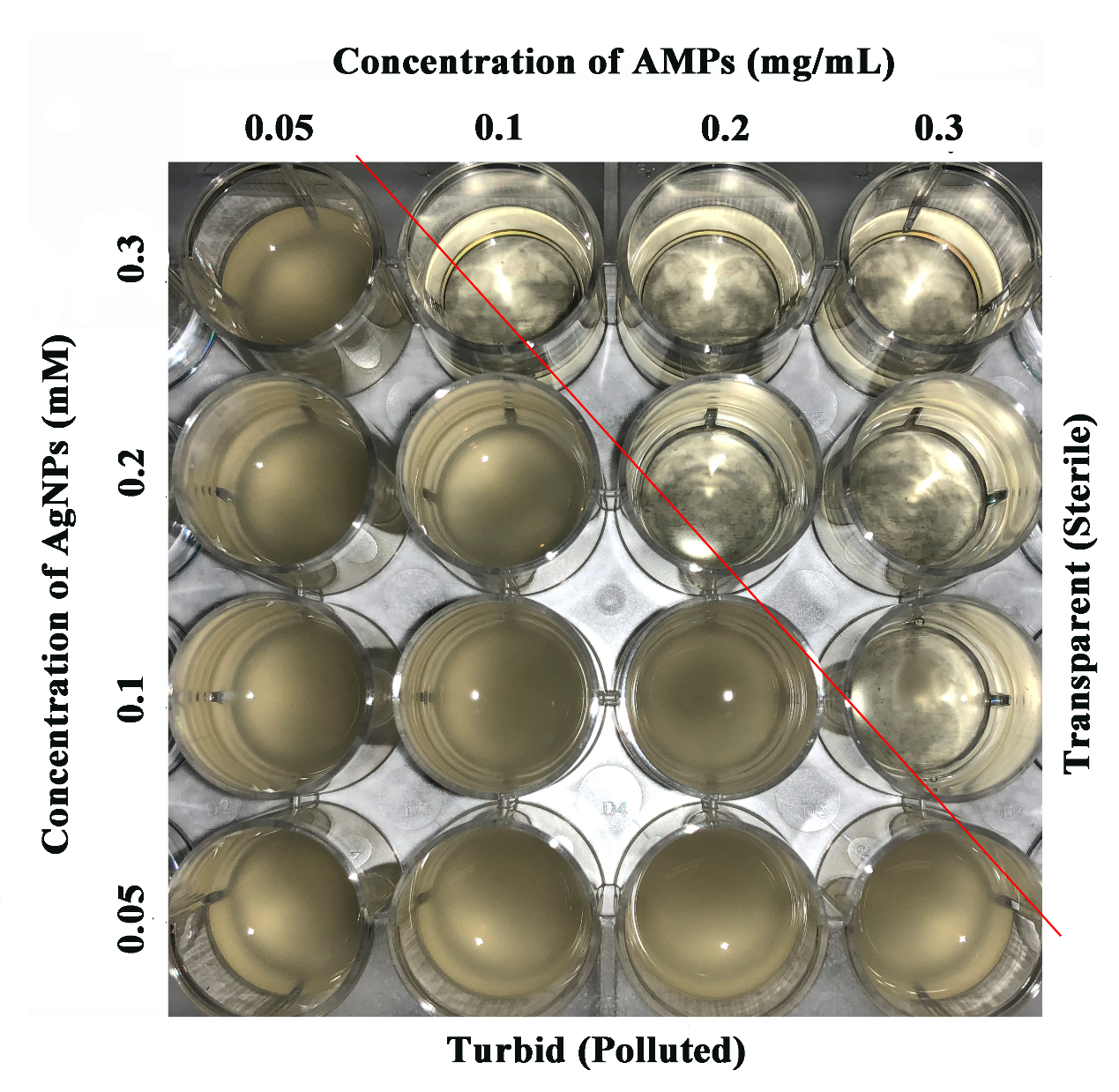


Figure S2. The synergistic antibacterial effect test of AgNPs/AMPs complexes, the survival of bacteria after co-cultivation with *Staphylococcus aureus* (1×10^6^ CFU/mL) for one day.


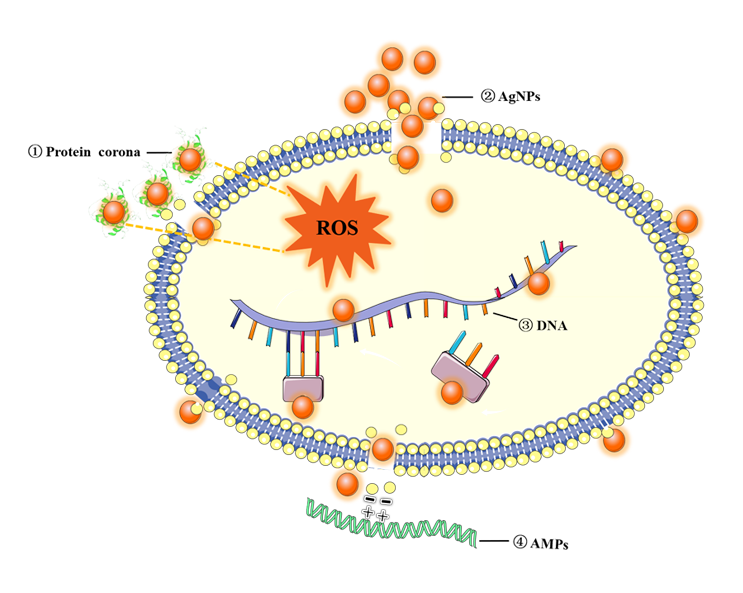


Figure S3. Schematic illustration of bacteria-triggered synergistic bactericidal mechanism of AgNPs/AMPs complexes.





Figure S4. Inhibition of the biofilm formation ability by crystal violet staining.


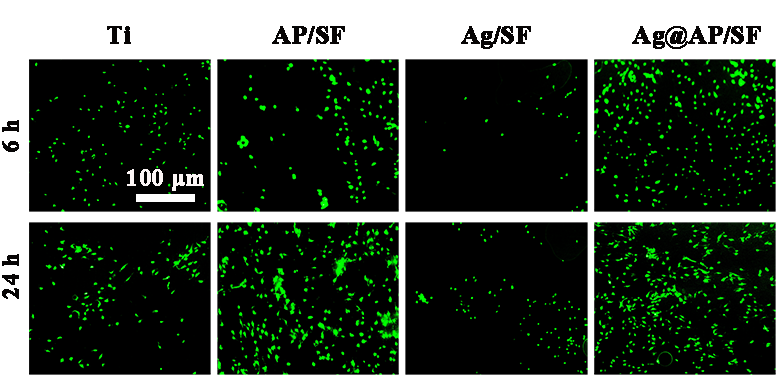


Figure S5. Live/Dead staining of BMSCs co-culture on the samples.
